# Production and characterization of rhamnolipid biosurfactant from thermophilic *Geobacillus stearothermophilus* bacterium isolated from Uhud mountain

**DOI:** 10.3389/fmicb.2024.1358175

**Published:** 2024-05-30

**Authors:** Hibah M. Albasri, Asmaa A. Almohammadi, Areej Alhhazmi, Duaa A. Bukhari, Moayad S. Waznah, Asmaa M. M. Mawad

**Affiliations:** ^1^Department of Biology, College of Science, Taibah University, Madinah, Saudi Arabia; ^2^Clinical Laboratory Sciences Department, College of Applied Medical Sciences, Taibah University, Madinah, Saudi Arabia; ^3^Botany and Microbiology Department, Faculty of Science, Assiut University, Assiut, Egypt

**Keywords:** antimicrobial activity, biosurfactants, *Geobacillus*, rhamnolipid, *rhl* gene, thermophilic bacteria

## Abstract

**Introduction:**

Biosurfactants have been given considerable attention as they are potential candidates for several biotechnological applications.

**Materials and methods:**

In this study, a promising thermophilic biosurfactant-producing HA-2 was isolated from the volcanic and arid region of Uhud mountain, Madinah, Saudi Arabia. It was identified using 16S rRNA gene sequence analysis. The biosurfactant production ability was screened using different methods such as the drop collapse test, oil spreading test, hemolytic activity test, CTAB test, and emulsification index. The ability of rhamnolipid production by the tested strain was confirmed by the polymerase chain reaction (PCR) of *rhlAB*. The affinity of thermophilic HA-2 to hydrophobic substrates was also investigated. Optimization of biosurfactant production was conducted. The biological activities of produced surfactant were investigated.

**Results and discussion:**

The isolated HA-1 was identified as *Geobacillus stearothermophilus* strain OR911984. It could utilize waste sunflower frying oil (WSFF) oil as a low-cost carbon source. It showed high emulsification activity (52 ± 0.0%) and positive results toward other biosurfactant screening tests. The strain showed high cell adhesion to hexane with 41.2% cell surface hydrophobicity. Fourier-transform infrared (FTIR) spectra indicated the presence of hydrophobic chains that comprise lipids, sugars, and hydrophilic glycolipid components. The optimization results showed the optimal factors included potato peel as a carbon source with 68.8% emulsification activity, yeast extract as a nitrogen source with 60% emulsification activity, a pH of 9 (56.6%), and a temperature of 50° (72%). The kinetics showed that optimum biosurfactant production (572.4 mg/L) was recorded at 5 days of incubation. The produced rhamnolipid biosurfactant showed high antimicrobial activity against some human and plant pathogenic bacterial and fungal isolates and high antioxidant activity (90.4%). In addition, it enhanced wheat (*Triticum aestivum*) growth, with the greatest enhancement obtained with the 5% concentration. Therefore, thermophilic *G. stearothermophilus* is a promising rhamnolipid biosurfactant producer that utilizes many organic wastes. The produced biosurfactant could be applied as a promising emulsifier, antimicrobial, antioxidant, and plant growth promoter.

## Graphical Abstract

The isolation of biosurfactant producing bacteria, gene detection and characterization of producing biosurfactant. 
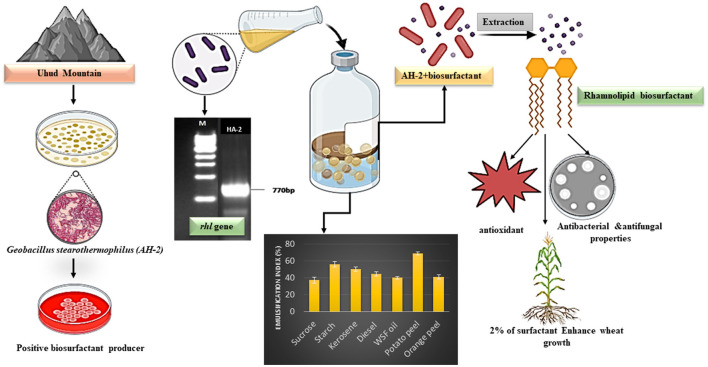

## 1 Introduction

Surfactants are molecules that possess both hydrophilic and hydrophobic groups, rendering them amphiphilic and capable of decreasing surface tension across various fluid phases (Ghosh et al., 2020). Compared to their industrially synthesized counterparts, biosurfactants demonstrate superior emulsification, detergency, and dispersion capacities, sparking considerable interest in their application across diverse sectors, including bioremediation, biodegradation, oil recovery, pharmaceuticals, food, cosmeceuticals, cleaning industries, and environmental safety (Gudiña et al., 2015; De Almeida et al., 2016; Paraszkiewicz et al., 2021). In recent times, biosurfactants represent only 10% of the world's total surfactant production, which is ~10 million tons per year (Silva et al., 2024). Microbial biosurfactants are chemicals produced by a wide range of microorganisms that have properties such as lowering surface tension, biodegradability, low toxicity, emulsion/de-emulsion, temperature, and pH tolerance (Roy, 2017). They improve the solubility of hydrophobic organic compounds, making them accessible for energy and carbon utilization (Kaczorek et al., 2018). The hydrophobic components of biosurfactants include long-chain fatty acids, and the hydrophilic components include carbohydrates, amino acids, or phosphate groups. The introduction of biosurfactants induces the solubility of the hydrophobic components within the lipid phase and the solubility of the hydrophilic components within the aqueous phase (Eswari et al., 2019). Because of the abovementioned mechanism of action, they perform an essential function in pollutant remediation at contaminated sites (Ng et al., 2022).

Different criteria can be used to distinguish various surfactants produced from microorganisms (Srivastava et al., 2022). However, in many literature studies, biosurfactants are primarily classified into low-molecular weight and high-molecular weight biosurfactants. The low-molecular weight group includes glycolipids, lipopeptides, and phospholipids, whereas the high-molecular weight group includes polymeric and particulate biosurfactants (Sharma and Sharma, 2016; Venkataraman et al., 2022). Rhamnolipids are a class of biosurfactants with unique characteristics that depend on the producer, substrate, and production conditions. They can significantly decrease the surface tension of water, making them effective at spreading and interacting with surfaces. The critical micelle concentration (CMC) also depends on the exact composition of the rhamnolipid mixture, typically falling within a range of 50–200 mg/L (Santos et al., 2016).

Thermophilic bacteria-produced biosurfactants have numerous advantages over the mesophilic ones. Elevated incubation temperatures mitigate the risk of contamination by additional pathogens, whereas the microorganism's high metabolic activity propels the reaction rate to a peak (Mehetre et al., 2019; Karadayi et al., 2021). Moreover, the viscosity of the production medium and cooling expenses are also diminished when using large fermenters in the production process (Eswari et al., 2019). Thermophilic *Bacillus licheniformis* B3–15 and *Bacillus horneckiae* SBP3 are biosurfactant-producing *Bacilli* collected from marine shallow-water hydrothermal vents (Zammuto et al., 2022). *Geobacillus* are spore-forming, Gram-positive, rod-shaped bacteria found in temperate and tropical habitats including hot springs, oilfields, deep sea sediments, sugar refineries, canned goods, and food processing factories (Burgess et al., 2017). *Geobacillus* species are aerobic or facultative anaerobic bacteria that thrive at 35–76°C in neutral or mild alkaline pH (Wada and Suzuki, 2020). They originally belonged to *Bacillus* Group 5 and were reclassified in 2001 (Nazina et al., 2001). The genus *Bacillus* is popular for bioremediation, production of thermostable enzymes, and biofuels (Feng et al., 2007; Bhalla et al., 2013). *Geobacillus stearothermophilus* was first isolated in 1920 from canned cream-style maize (Zarilla and Perry, 1987).

Many biosurfactants have strong antibacterial and antifungal activities and play a significant role as anti-adhesive agents to pathogens, which make them effective in treating a wide array of diseases (Rodrigues et al., 2006; Gudiña et al., 2015; De Giani et al., 2021). They are promising biomolecules for the agricultural sector (Silva et al., 2024). Fortunately, few research studies have mentioned the direct impact of produced and extracted biosurfactants on wheat plant growth.

Uhud mountain is one of Arab Peninsula's significant historic and arid locations and can be observed across the other mountains of this area. It is 1,077 m high, 7 km long, and 3 km wide, forming a chain from east to west. The mountain has some of the most stunning granite rock formations, which are dark red in color and include many mineral veins of various colors, such as blue, black, white, green, and silver (Obaid et al., 2020). Interestingly, there are no reports studying microbial habitats and their applications on this mountain to date.

Therefore, this study aimed to screen, isolate, and identify thermophilic biosurfactant-producing bacteria from soil in Uhud mountain, Madinah, Saudi Arabia; to investigate the type of produced biosurfactants; to optimize biosurfactant production; and to determine the role of the produced biosurfactants on bioactivity in pathogenic microorganisms and one crop seed germination.

## 2 Materials and methods

### 2.1 Sample collection

Soil samples were collected from the north of Uhud mountain located at 24°32′42.6^′′^N, 39°36′36.2^′′^E and at a depth of 10–15 cm from the soil surface. The samples were placed in sterilized plastic bags and transferred directly to the laboratory for further experiments.

### 2.2 Enrichment and isolation of biosurfactant-producing bacteria

Mineral basal medium “MBM” was prepared and used to isolate and enrich bacteria that produce biosurfactants. The medium consists of (g/L): 1.0 (NH_4_)_2_SO_4_, 0.8 K_2_HPO_4_, 0.2 KH_2_PO_4_, 0.2 MgSO_4_·7H_2_O, 0.1 CaCl_2_·2H_2_O, and 0.005 FeSO_4_·7H_2_O. After adjusting the pH to 7.0 ± 0.2, it was autoclaved at 121°C for 20 min (Hesham et al., 2014). A waste sunflower frying oil (WSFF; 10%) was supplemented to the autoclaved MBM as the sole source of carbon after sterilization utilizing a filter unit with a pore size of 0.2 μm (Millipore Crop., Bedford, MA, USA). The soil sample 10% (w/v) was then added to the previous mixture, incubated at 50°C, and centrifuged at 120 rpm in an orbital shaker for 7 days. Following incubation, a 10% aliquot was transferred into 90 mL of fresh autoclaved MBM supplemented with sterilized oil as previously described and incubated in an orbital shaker at 50°C for another 7 days. This step was repeated three times for enrichment oil adaptation and biosurfactant-producing bacteria.

For the isolation of thermophilic bacteria, the MBM sterilized agar plates were coated with sterilized WSFF oil and inoculated with 100 μL of the enriched culture, and then, they were incubated at 50°C until the growth of oil-emulsifying bacteria colonies. The colonies then were separately selected, purified using the streak plate method, and the pure colony was tested for oil emulsification activity. The colony with a high emulsification index was selected for further characterization.

### 2.3 Screening of potent biosurfactant-producing isolates

The qualitative and quantitative characterization of biosurfactant activity was performed by using various tests, including the emulsification index (E_24_) test, hemolytic test, cetyltrimethylammonium bromide (CTAB) test, oil displacement test, and drop-collapsing test. All experiments were performed in triplicate using thermophilic bacterial cell-free extract. Positive controls including sodium dodecyl sulfate (SDS; Thermo Scientific, USA) and CTAB (Sigma-Aldrich, USA) were used, while MBM was used as a negative control.

#### 2.3.1 The emulsification index

The emulsification activity of the produced biosurfactant was assessed in accordance with the guidelines provided by Ismail et al. (2018). A volume of 2 mL of oil was added to an equal amount of the cell-free supernatant in a test tube. Then, the tube was vortexed for 10 min at high speed and allowed to stand for 24 h. The emulsification index (E_24_) was calculated as the ratio of the emulsion layer's height to the total height of the liquid, as given by the following Equation 1:


(1) $$\begin{array}{l} {\text{E}}_{24}\left(\%\right)=\left(\text{The height of emulsion layer}\right)\\ /{\left(\text{The total height of the liquid column}\right)}^{*}100 \end{array}$$

#### 2.3.2 The Biuret test

This test was applied to detect lipopeptide biosurfactants. A volume of 2 mL of the extracted surfactant was mixed with 1 M NaOH solution. Then, 1% of CuSO_4_ was added dropwise gradually. The appearance of a violet or pink ring was considered to be a positive result, indicating the reaction between short-chain polypeptides or peptide bond proteins (Jamal et al., 2012).

#### 2.3.3 Hemolytic test

An aliquot of 100 μL of the extracted biosurfactant was spot-inoculated onto a well on the blood agar plate (S.G.H) and incubated at 37°C for 24–48 h, and the hemolytic clear halos around the well-indicated a positive response (Bharali et al., 2011).

#### 2.3.4 CTAB test

CTAB agar (Magen, China) was prepared by adding 0.15 g of CTAB, 0.005 g of methylene blue (Sigma-Aldrich, USA), and 12 g of agar to 1 L of distilled water. After sterilization, holes were made in CTAB agar plates, and a volume of 100 μL of the extracted biosurfactant was loaded into the hole. The plates were incubated at 37°C for 24–48 h. Dark blue zones observed around the wells indicated anionic biosurfactant production (Rani et al., 2020).

#### 2.3.5 Drop collapsing test

A modified drop-collapse method was implemented by utilizing 96-well microtiter plates containing 100-μL oil stained with Sudan III (HIMEDIA, USA) and was left for 24 h at room temperature. A volume of 50 μL of the extracted biosurfactant was added to the surface of the well, and the drop size was measured after 1 min by using a magnifying glass. The production of biosurfactants was deemed positive when the droplet diameter was 1 mm greater than the diameters produced by the culture medium and distilled water, which served as negative controls (Jain et al., 1991; Ahmad et al., 2016).

#### 2.3.6 Oil spreading test

This method was employed to evaluate the effectiveness of the culture medium in displacing the oil layer. Approximately 2 mL of oil stained with Sudan III was added to the surface of 30 mL distilled water in a Petri dish to form a thin oil layer. A volume of 20 μL of the extracted biosurfactant was gently added on the center of the oil layer. The oil displacement indicated positive surfactant activity, and the clearing zone was measured (Sidkey et al., 2016).

#### 2.3.7 Bacterial adhesion to hydrocarbon (BATH) assay

This assay, as described by Sumathi and Yogananth (2016), was used to determine the bacterial cell surface hydrophobicity. The thermophilic bacterial isolates were separately grown on nutrient broth supplemented with oil and incubated at 50°C with orbital shaking at 150 rpm for 72 h. After incubation, the bacterial culture was centrifuged at 4,000 rpm for 10 min. The pellet was washed twice with phosphate-buffered saline (PBS) and then suspended in 2.6 mL of the buffer to give initial OD_600_ = 0.3. Then, a volume of 400 μL of hexane (EMPLURA^®^ Merck, Germany) was added to the cell suspension and vortexed for 3 min. The hexane and aqueous phases were allowed to separate for 1 h. The optical density of the aqueous phase was then measured at 600 nm using a spectrophotometer (APEL, PD-303UV). The hydrophobicity (H%) was expressed as the percentage of cell adherence to hexane and calculated as shown in Equation (2).


(2) $$\begin{array}{l} \text{H}\%=\left({\text{InitialOD}}_{600}-{\text{FinalOD}}_{600}\right)/{\left({\text{InitialOD}}_{600}\right)}^{*}100 \end{array}$$

### 2.4 Molecular identification of the potent biosurfactant-producing isolate

For molecular identification, gDNA was extracted from the pure isolate using the PrepManPureLink™ Genomic DNA Mini Kit (Invitrogen) according to the manufacturer's protocol by the Macrogen company in Seoul, South Korea. For identification, universal bacterial primers 27F ^5^′(AGA GTT TGA TCM TGG CTC AG)^3^′ and 1492R ^5^′(TAC GGY TAC CTT GTT ACG ACT T)^3^′ were used to amplify the 16S rRNA region. Axen^TM^ H Taq PCR Master Mix (2X) was used for amplification according to protocol of Macrogen Polymerase Chain Reaction (PCR) system cycler. The reaction program consists of denaturation at 94°C for 5 min, 31 cycles of amplification performed by denaturing for 0.5 min at 95°C, annealing for 0.5 min at 57°C, and extension for 1.4 min at 72°C. The PCR products were sequenced in both directions using an ABI 3730 automated sequencer by Macrogen (Seoul, Korea). The sequences obtained were compared to established 16S rRNA gene sequences in the GenBank database via the basic local alignment search tool (BLAST) at the National Center for Biotechnology Information (http://www.ncbi.nlm.nih.gov/BLAST/), and percent homology scores were computed in order to ascertain the bacterial isolate. MEGA version 4.0 was utilized to construct the phylogenetic tree via the Jukes–Cantor distance estimation and the neighbor-joining method, with 1,000 bootstrap replicates, to calculate the confidence values among species (Hesham and Mohamed, 2011).

### 2.5 Optimization of biosurfactant production

A series of experiments were performed to determine the optimum conditions in changing one variable at a time. These factors include carbon and nitrogen sources, pH, and incubation time. A volume of 1% (v/v) inoculum of 24-h-old freshly grown culture (OD_600_ = 0.23) was used in all the experiments. Bacterial cells were removed by centrifugation at 5,000 rpm for 15 min. Production of biosurfactants was evaluated in terms of the emulsification index. The impact of pH was determined by justifying different pH values (5–9) by using HCl or NaOH (1N). For determination of the impact of nitrogen sources, ammonium sulfate was eliminated from the MBM and replaced by 1.0 g/L of either yeast extract, peptone, beef extract, or sodium nitrate (Parthipan et al., 2017).

#### 2.5.1 Emulsification index on different organic substrates

At the optimum production conditions, the isolate was evaluated to determine its capability to produce biosurfactants in the presence of different wastes. Six organic wastes (1%), namely, starch, kerosene, diesel, WSFF oil, potato peel, and orange peel, were separately supplemented into MBM sterilized media as the sole source of carbon. Sucrose (Merk, Germany) served as a standard carbon source. The potato and orange peels were oven-dried at 70°C and aseptically crushed into powder using a grinder. The emulsification index was determined as per the previously described method, and sucrose was used as a control.

### 2.6 Extraction and quantification of produced biosurfactants

The pure bacterial isolates were inoculated onto MBM with sterilized waste frying oil and incubated at 50°C with 120 rpm shaking for 15 days. After incubation, the culture was centrifuged (MPW-350R) at 5,000 rpm for 15 min to remove the bacterial cells. Then, 6 N HCl was added to the supernatants for precipitation until a pH of 2.0 is reached. The acidified supernatants were kept overnight at 4°C. The precipitated biosurfactant was extracted with an equal amount of chloroform–methanol (2:1 v/v; EMPLURA^®^ Merck, Germany) mixture and concentrated by evaporation. The extracted biosurfactants were used in further experiments (Kumar et al., 2016).

For quantification of rhamnolipids, distilled water was utilized to solubilize the crude extract, and the quantity of rhamnolipids was determined using the phenol–sulfuric acid method, with rhamnose serving as the standard. A volume of 0.5 mL of the supernatant was added to 0.5 ml of 5% phenol solution and 2.5 mL of sulfuric acid (Scharlau, Spain). The mixture was incubated for 15 min, and the absorbance was measured at 490 nm by using a spectrophotometer (Sidkey et al., 2016). The association between rhamnose and rhamnolipids is illustrated by the commonly used method of multiplying the quantity of rhamnose by a factor of 3.4 in order to ascertain the concentration of rhamnolipids generated by thermophilic bacteria (Raza et al., 2014; Safari et al., 2023).

### 2.7 Kinetics of biosurfactant production by the thermophilic isolate

The kinetics of surfactant production by thermophilic bacteria were determined at optimum conditions. The MBM supplemented with the optimum carbon source (sucrose) was inoculated with 2% (v/v) of 48-h pre-grown cells of thermophilic bacteria. It was incubated at 50°C for 2–9 days. The samples were withdrawn every day, and the quantity of the biosurfactants was determined. The kinetics parameters were cell biomass (g/L), emulsification index (%), and biosurfactant concentration (g/L; Abbasi et al., 2012). To determine the dry biomass (g/L), the grown bacterial cells were centrifuged at 10,000 × *g* for 15 min, the pellets were washed twice with water, oven-dried at 105°C overnight, and weighted. The concentration of biosurfactants as well as the emulsification index (%) were determined as previously described.

### 2.8 Determination surface tension and critical micelle concentration

The surface tension (mN/m) of the produced biosurfactant solution was determined using a stalagmometer for the drop counting method (Kaya et al., 2014; Joe et al., 2019). The surface tension was estimated using Equation (3) as the follow:


(3) $$\begin{array}{l} {\text{σL=σW}}^{\text{*}}{\text{Nw/NL}}^{\text{*}}\text{ρL/ρW} \end{array}$$

where σL and σW are the surface tension of biosurfactant solution and water, respectively. NL and NW are the drop number of the biosurfactant solution and water, respectively. ρL and ρW are the density of the biosurfactant solution and water, respectively.

To determine the critical micelle concentration (CMC), the aqueous biosurfactant solution was serially diluted (500–0 mg/L), and the surface tension values of these dilutions were obtained. Experiments were performed in triplicates at room temperature, and mean values were recorded. The CMC of the biosurfactant solution was obtained from the breakpoint of the surface tension plotted vs. the biosurfactant concentration (Kaya et al., 2014; El-Housseiny et al., 2020).

### 2.9 Detection of gene encoding enzymes implicated in biosurfactant synthesis

The rhamnosyl transferase (*rhlAB*) gene was amplified using rhlABF ^5^′(CAG GCC GAT GAA GGG AAA TA)^3^′ and rhlABR ^5^′(AGG ACG ACG AGG TGG AAA TC)^3^′ primers (Kumar et al., 2008). The PCR was conducted using a mixture containing 1 μL of the DNA template of the selected biosurfactant producer. Each primer (0.2 μM) includes 10 × reaction buffer, MgCl_2_ (1.5 mM), dNTPs (200 μM), and Taq DNA (1 U) polymerase in a Thermal Cycler (BioRad). DNA was amplified at 95°C for 5 min, followed by 30 cycles of denaturation for 30 s at 95°C, annealing for 1 min at 50°C, and extension for 2 min at 72°C, and a final extension step for 10 min at 72°C (Pacwa-Płociniczak et al., 2014).

### 2.10 Characterization of the produced biosurfactant

Thin-layer chromatography (TLC) was employed to determine the chemical nature of the biosurfactant; ~4 μL of the extracted biosurfactant was dissolved in chloroform: methanol: water (6.5:1.5:0.2 v/v) and loaded onto a TLC plate (sigma-Aldrich, Germany) using methanol as a mobile phase. Then, the plates were separately analyzed using iodine (Kamat and Kawale, 1987) and Molisch reagent (α-naphthol in ethanol with 10% H_2_SO_4_) for lipid and sugar detection, respectively. The formation of yellow brown spots indicates the reaction of iodine with lipids, while greenish–blue spots indicate the presence of sugar. The Rf values were then calculated (Lotfabad et al., 2010). The standard of rhamnolipid (AGAE technologies, USA) was used for comparison.

The Fourier-Transform Infrared (FTIR) spectrometer was employed to ascertain the occurrence of rhamnolipids in the bacterial cell-free extract. A volume of 3 mg of the extracted rhamnolipids was analyzed in the wavenumber range of 4,000–500 cm^−1^ using an FT-IR spectrometer.

### 2.11 Biological activity of the produced biosurfactant

#### 2.11.1 Antimicrobial activity

The extracted biosurfactant was screened for its antimicrobial activity against several human pathogenic bacterial strains including *Pseudomonas aeruginosa, Escherichia coli, Bacillus cereus*, and *Staphylococcus aureus* and plant pathogenic fungal strains such as *Aspergillus niger, Alternaria alternata, Fusarium solani*, and *Penicillium sp*., using the agar well diffusion method (500 μL of the biosurfactant per well). The assay was performed on sterilized Muller–Hinton agar (MH) and Sabouraud dextrose agar (SA) for the antibacterial and antifungal assays, respectively. The plates were incubated at 37°C for 24 h and at 25°C for 2–5 days for bacteria and fungi, respectively. After incubation, the clear zone diameter was measured (Garg and Chatterjee, 2018). Three independent experiments were performed, and the average was estimated.

#### 2.11.2 Antioxidant activity

The antioxidant activity was assessed using the 2,2-diphenyl-1-picryl-hydrazylhydrate (DPPH) free radical assay. The mixture was prepared by adding 500 μL of the supernatant, 300 μL of DPPH, and 3 mL of absolute ethanol. Then, the mixture was incubated in the dark at room temperature for 24 h. Color change from violet to yellow was monitored by determining the absorbance at 517 nm using a spectrophotometer. A sample of water and ethanol was used as the blank control, while ethanol and DPPH solution was used as the negative control (Mawad et al., 2018).

#### 2.11.3 Activity on plant growth

The effect of biosurfactants on plant growth was investigated. Ten minutes after being surface-sterilized with 5% sodium hypochlorite, the grains of *Triticum aestivum* (wheat) landrace Qasimi were washed three times with sterile water. Sterilized seeds (20 seeds) were placed on a cotton mesh moistened with 5 mL of the biosurfactant of the isolates at concentrations of 2, 5, and 10% in plastic boxes and incubated for 7 days. After incubation, the mass, radical length, coleoptile length, and longest seminal roots of grown plants were measured. Sterilized seeds incubated on cotton mesh moistened with sterile distilled water or MBM were used as controls (Marchut-Mikołajczyk et al., 2021).

### 2.12 Statistical analysis

The mean standard deviations of the results from three independent experiments were calculated and analyzed using a one-way ANOVA, followed by grouping information using the Fisher LSD method and a 95% confidence interval with Minitab statistics software version 19.0. *P*-values < 0.05 were deemed to be statistically significant.

## 3 Results

### 3.1 Enrichment and isolation of thermophilic biosurfactant-producing bacteria

Thirteen thermophilic biosurfactant-producing bacteria were isolated after enriching three times in MBM supplemented with WSFF oil as a sole source of carbon at 50°C for 7 days. One promising biosurfactant producer AH-2 showed a significant capability of emulsification on oil. Therefore, it was selected for further investigations.

For preliminary screening of biosurfactant production, the ability of the cell-free extract to emulsify oil was investigated. The AH-2 extract could emulsify oil with 52 ± 0.0%. In addition to the emulsification, it exhibited a positive hemolytic activity by formation of a clear halo zone. The positive response to the CTAB assay was confirmed by formation of a blue halo around the extracted surfactant. Additionally, the drop collapse value of the isolate HA-2 cell free extract was 4.63 ± 0.15 cm. The biosurfactant produced from HA-2 showed the highest oil displacement diameter of 5.2 ± 0.2 cm. Furthermore, the degree of the adherence of AH-2 cells to liquid hydrocarbons indicates increasing cell surface hydrophobicity. The percentage of hydrophobicity of the cells was 41.2% (Table 1). It was noticed that the results of the biuret test showed no change in color, which indicated a negative response and non-amino acid moiety of the produced surfactant.

**Table 1:** Biosurfactant activity assays and HA-2.

**Treatment**	**Emulsification index (%)**	**Hemolysis**	**Drop collapse (cm)**	**Oil spreading (cm)**	**CTAB**	**BATH %**
HA-2	52 ± 0.0	+	4.63 ± 0.15	5.2 ± 0.2	+	41.2
CTAB	63 ± 0.0	ND	5.16 ± 0.1	5.2 ± 0.1	ND	ND
MBM	0	-	0.2 ± 0.0	0.0 ± 0.0	-	ND
SDS	ND	+	ND	ND	+	ND

CTAB (positive control); MBM (negative control); SDS (positive control); +, - positive and negative responses. All values are represented as mean ± standard deviations from triplicate experiments.

ND, not determined.

### 3.2 Molecular genetic characterization

**Figure 1:**
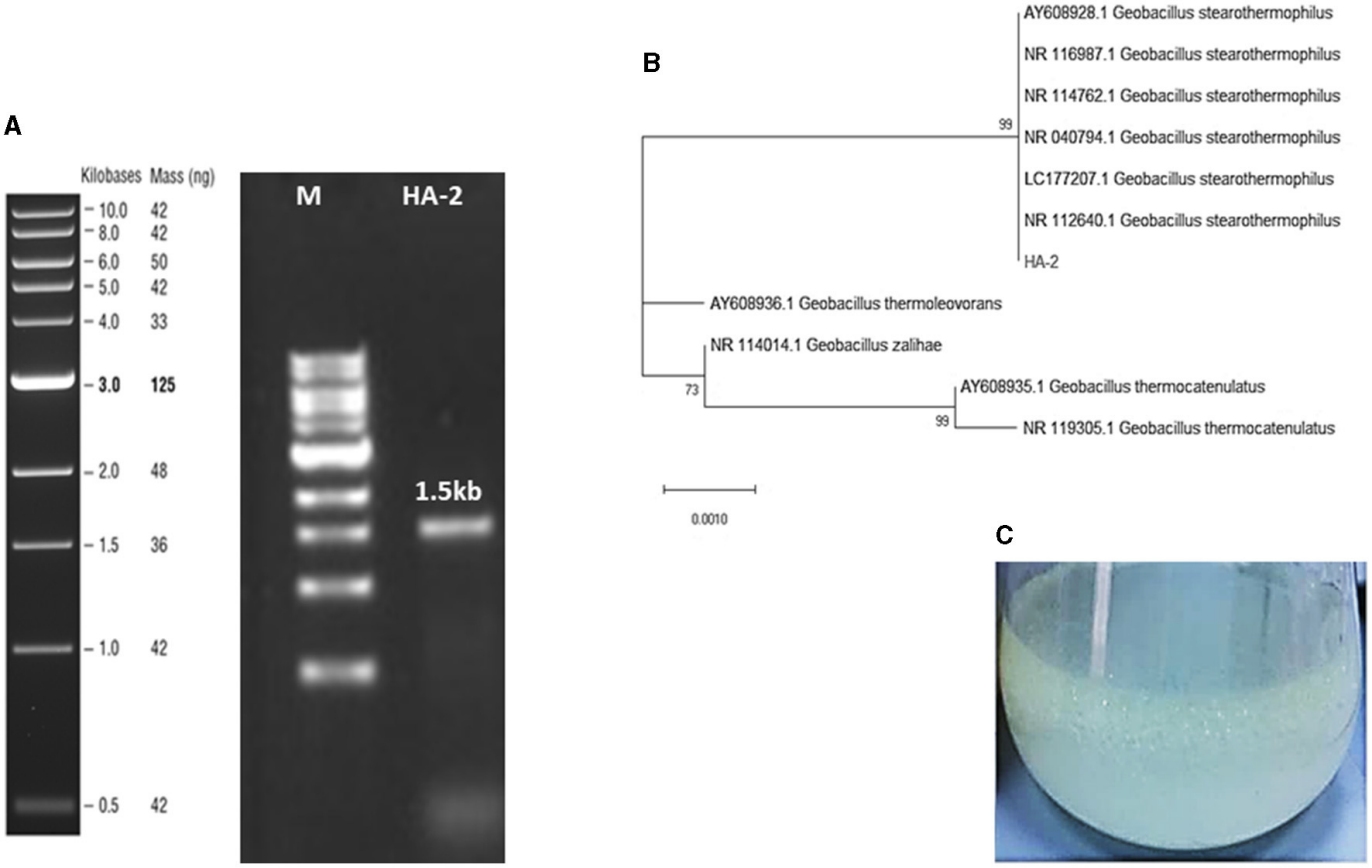
**(A)** Agarose gel stained with ethidium bromide resolved the 1.5-Kb PCR-amplified fragment of the 16S rRNA gene sequence from the AH-2 isolate. **(B)** The maximum likelihood phylogenetic tree constructed based on 16S rRNA gene sequences showed the association between strain HA-2 and the closely related strains within the genus *Geobacillus*. Bootstrap values (expressed as percentages of 1,000 replications) of more than 50% are shown at the branch points. The scale bar indicates 0.01. Substitutions per nucleotide position. **(C)** WSFF oil emulsification and biosurfactant production in the MBM at 50°C.

The sequence of the 16S rRNA gene of the isolated bacteria HA-2 demonstrated a high degree of similarity (99%) with the *Geobacillus stearothermophilus* strain BGSC 9A20 (Figures 1A, B). The 16S rRNA sequences of the strain were determined and submitted to the GenBank database with accession number OR911984 (HA-2).

### 3.3 Concentration of the biosurfactant

The results obtained indicated that a rhamnolipid concentration of 413.38 mg/L could be produced after a period of 7 days using WSFF oil as a sole carbon source (Figure 1C).

### 3.4 Optimization of biosurfactant production from thermophilic AH-2

#### 3.4.1 Impact of pH and temperature on biosurfactant production

**Figure 2:**
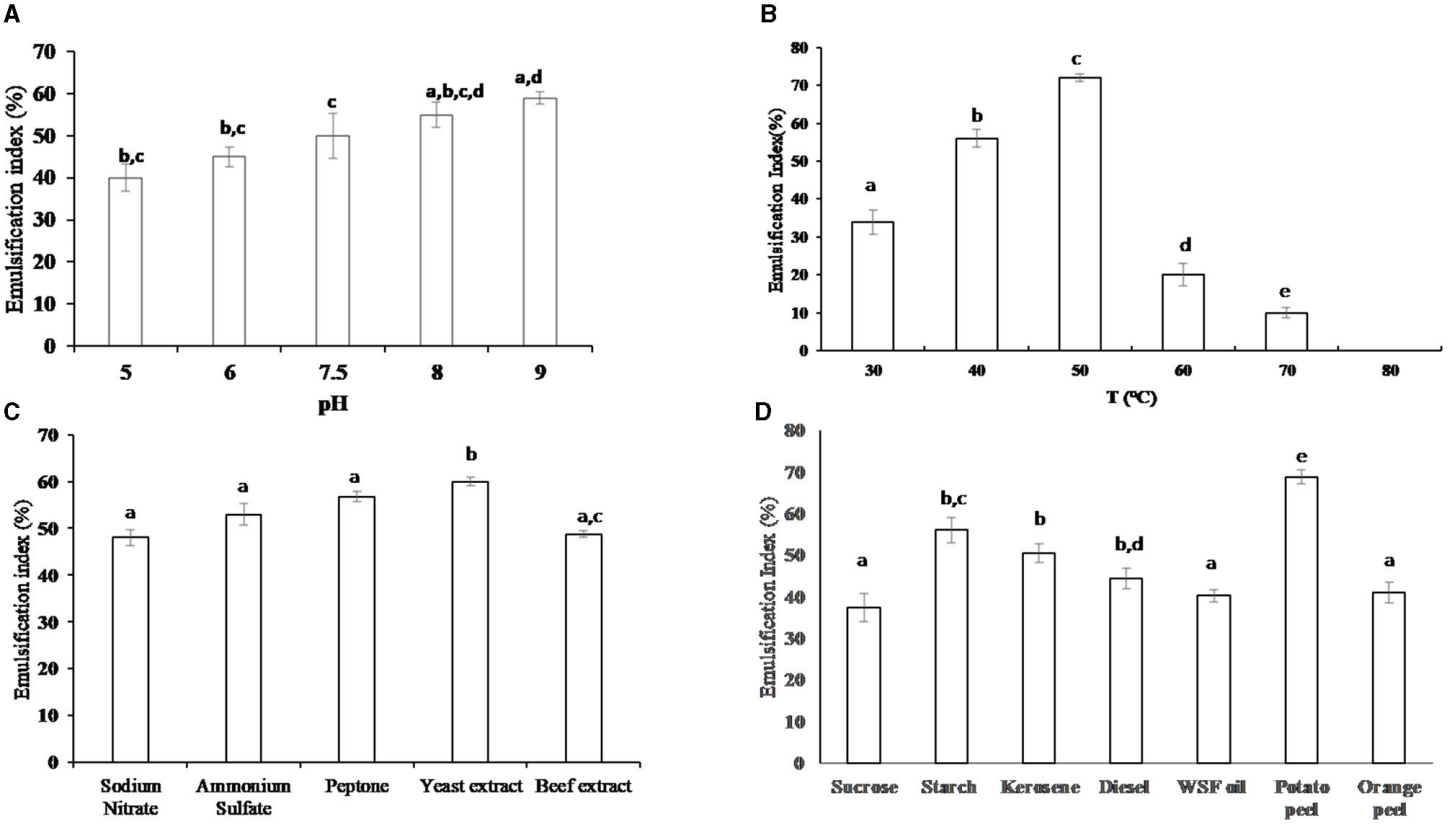
The emulsification index (%) of the produced biosurfactant from the cells of thermophilic AH-2 at different environmental and nutritional conditions; **(A)** impact of pH, **(B)** impact of temperature, **(C)** impact of different nitrogen sources (1%), and **(D)** impact of different organic carbon sources (1%). Plotted values are the means of triplicate treatments ± standard deviation of the mean. The impact of the revelators corresponding to each treatment and not sharing the same letters are significantly different according to the Fisher LSD method and 95% confidence intervals.

Different pH values (5, 6, 7.5, 8, or 9) were adjusted to determine the impact of pH on the biosurfactant emulsification index. The optimum emulsification index (59.7%) was estimated at pH = 9. The results in Figure 2A reveal that the bacterial isolate could produce and emulsify WSFF oil in alkaline and acidic media; however, the emulsification indices 55 and 59.7% in alkaline media pH of 8.0 and 9.0, respectively were significantly (*P* ≤ 0.05) higher than the emulsification indices 40 and 45% in acidic media pH of 5.0 and 6.0, respectively.

Regarding the effect of incubation temperature on the emulsification index, the thermophilic bacterial isolate exhibited emulsification activity at various temperature ranges from 30 to 70°C; however, the optimum emulsification index (72%) was significantly (*P* ≤ 0.01) recorded at 50°C when compared with the investigated temperature, as illustrated in Figure 2B. No emulsification activity of the bacteria was observed at 80°C when compared with the control sample at the same temperature.

#### 3.4.2 Impact of nitrogen and organic substrates on emulsification activity

The impact of inorganic and organic nitrogen on the production of biosurfactants was investigated as shown in Figure 2C. The results indicate that there was no significant variation between the emulsification index in the presence of various nitrogen sources. However, the beef extract exhibited a significantly (*P* ≤ 0.05) lower emulsification index (48.76%) when compared with the emulsification indices 60, 56.8, and 53.3% in the presence of yeast extract, peptone, and NH_4_(SO_4_)_2_, respectively. On the other hand, yeast extract is an optimum nitrogen source that induced an optimum emulsification index of 60%.

Concerning the influence of organic carbon on the production of biosurfactants, the results in Figure 2D showed that potato peel was the optimum carbon source that induced highest (*P* ≤ 0.05) biosurfactant production (68.79% of emulsification index) when compared with the other investigated substrates. The emulsification indices for starch, kerosene, and diesel were 56, 50.5, and 44.3%, respectively. It was noticed that there was no significant difference between the values of emulsification index when orange peel and WSFF oil were used. On the other hand, sucrose exhibited the lowest value of emulsification index (37.3%).

### 3.5 Kinetics of biosurfactant production

**Figure 3:**
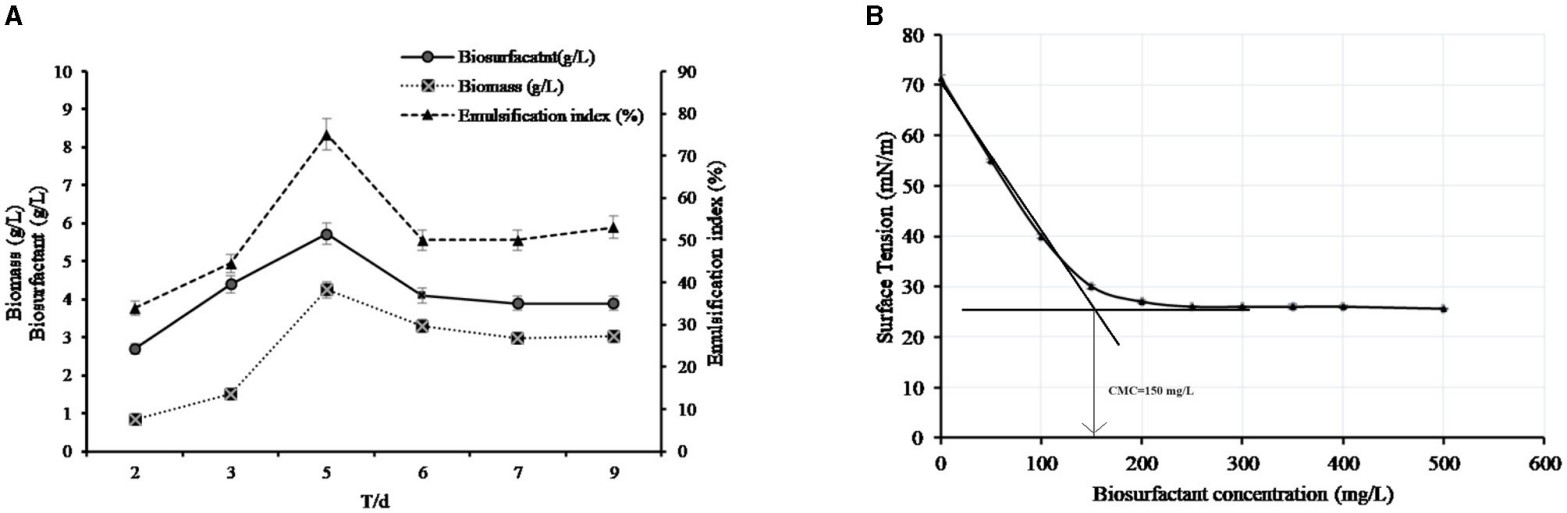
**(A)** Kinetics of the production of biosurfactants by AH-2 in MBM with 10 mL of the initial volume of the bacterial culture at 50°C. **(B)** Critical micelle concentration of the biosurfactant. The plotted values are the means of triplicate treatments ± standard error of the mean.

During determination of the biosurfactant concentration for a period of 9 days of cultivation, the level of biosurfactant concentration initially increased linearly from 2 to 5 days, reaching a maximum production of 0.574 g/L at the 5th day of cultivation. It was noticed that, at the 6th day of cultivation, the amount of biosurfactants produced decreased to 0.41 g/L, and then, it achieved a plateau of ~0.39 g/L from 7 to 9 days of cultivation, as shown in Figure 3A. It was noticed that the biosurfactant production and emulsification index were growth-associated since the bacteria-derived biosurfactants during the early growth phase achieved the maximum concentration at the end of the exponential phase (4.256 g/L of biomass) with the highest emulsification index (75%). The stationary phase was observed at the 6th day of incubation with no extra biosurfactant production, and an emulsification index of 50% was recorded during this phase.

### 3.6 Surface tension and CMC

The produced biosurfactant solution could lower the surface tension of water from 71.23 ± 0.67 to 25.6 ± 0.1 mN/m. The CMC was calculated from a plot between biosurfactant concentration and surface tension that is measured at each concentration. The intersection of the plot in Figure 3B indicated that the CMC of the produced biosurfactant was 150 mg/L. The surface tension at the CMC was 30 mN/m (Figure 3B).

### 3.7 Detection of the *rhlAB* gene in the biosurfactant-producing thermophilic bacteria

**Figure 4:**
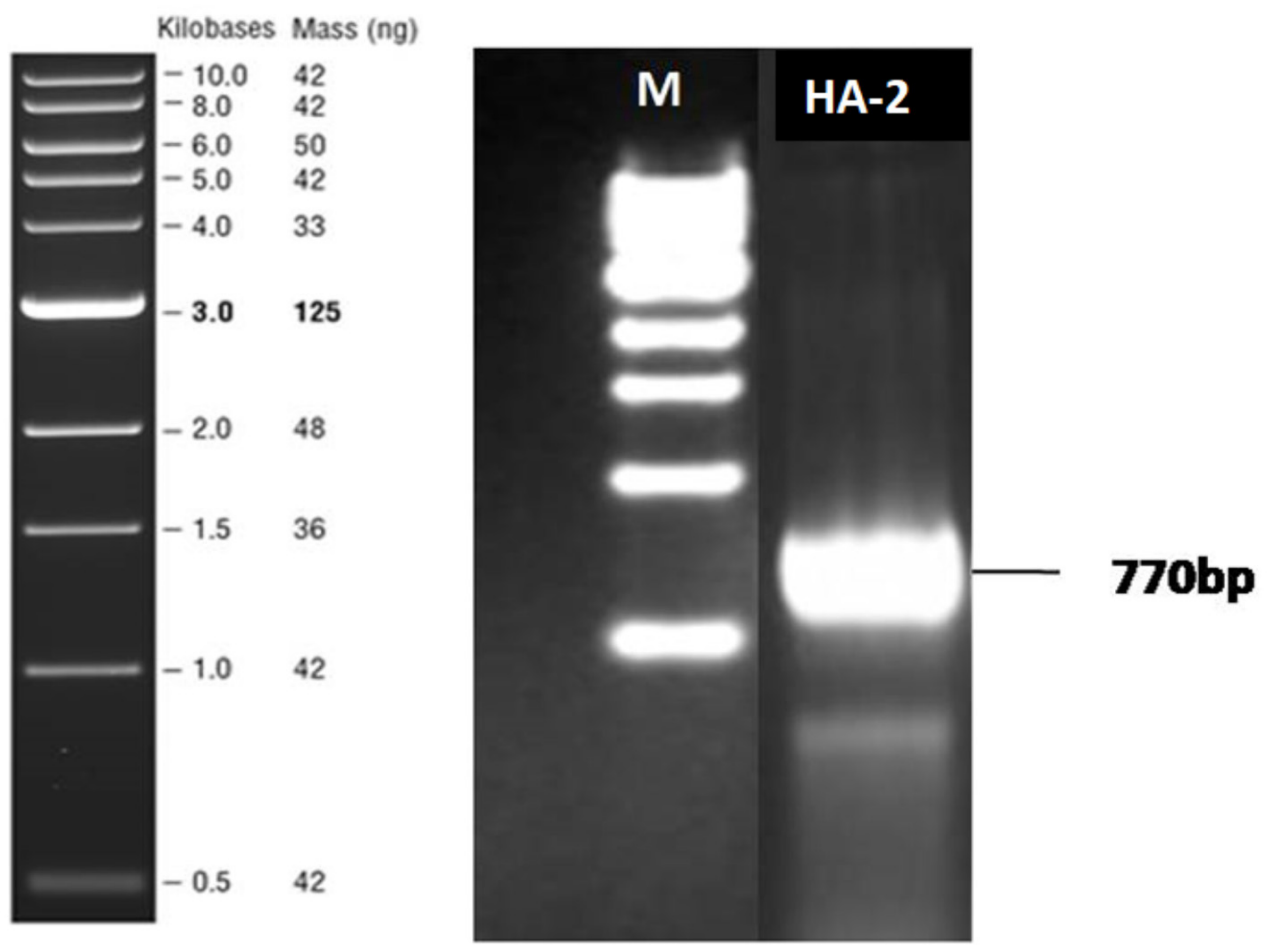
Agarose gel electrophoresis analysis after *rhlAB* amplification from the bacterial isolates. PCR-amplified products were run on 1% agarose gel. Lane M indicates 1-kb DNA ladder. Lane 1 indicates the PCR products (770 bp) of the respective bacterial isolate HA-2.

The ability of rhamnolipid synthesis by HA-2 was determined by the polymerase chain reaction of the *rhlAB* gene. The size of the DNA product detected was 777 bp in the thermophilic isolate when the primers *rhlAB*F and *rhlAB*R were used, as shown in Figure 4.

### 3.8 Chemical characteristics of rhamnolipid biosurfactant by FTIR analysis

**Figure 5:**
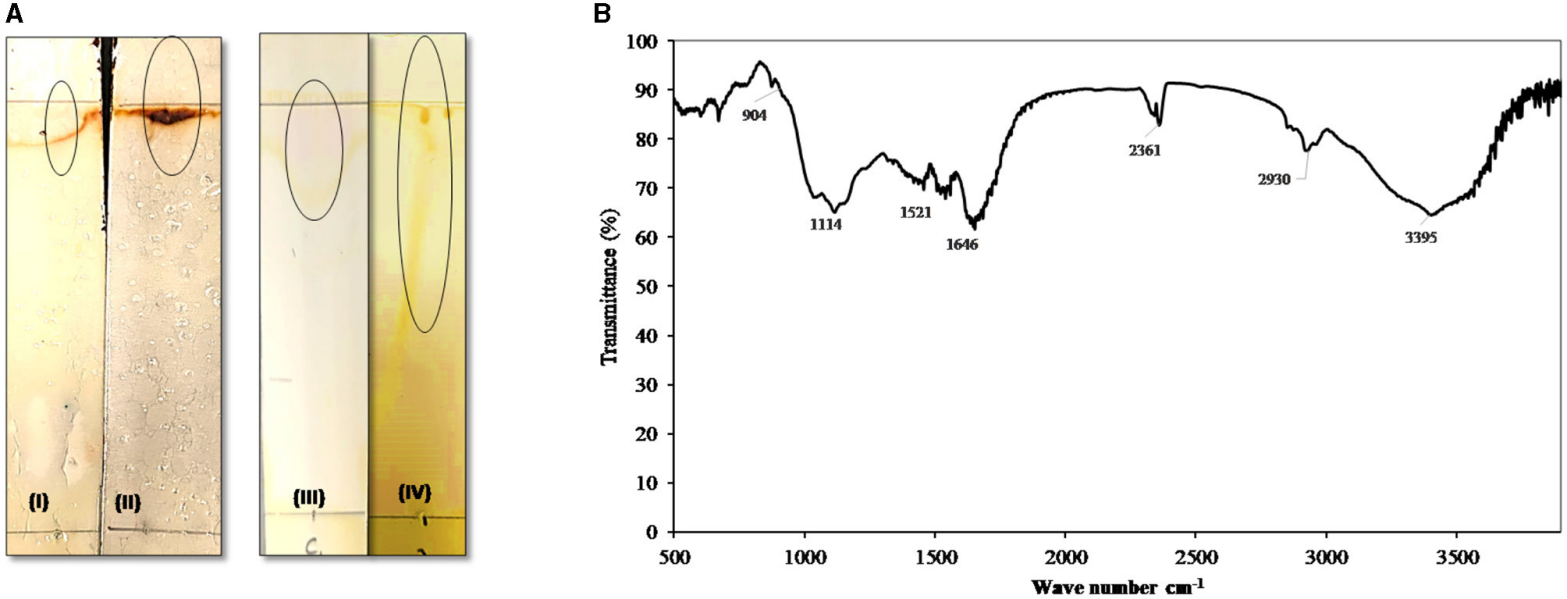
**(A)** Thin-layer chromatography (TLC): TLC plate run in a solvent system of chloroform: methanol: acetic acid (6.5:1.5:0.2), developed with Molisch reagent (I, standard and II, AH-2 surfactant) showing typical brown color spots of rhamnolipids and with iodine vapor (III, standard and IV, AH-2 surfactant) developed yellow color. **(B)** FTIR spectrum of the biosurfactants produced by AH-2 on waste sunflower frying oil.

Thin-layer chromatography (Figure 5A) of the extracted biosurfactant produced from thermophilic AH-2 showed one characteristic pot corresponding to the standard rhamnolipid surfactants on the reaction with Molisch reagent with a retardation factor (Rf) value of 0.89, confirming the presence of rhamnolipid surfactants. The positive reaction with iodine vapor indicated the presence of lipids in the rhamnolipid surfactant structure.

The functional groups of the produced biosurfactant were analyzed by FTIR spectroscopy (Figure 5B). The FTIR pattern showed that the broad band at regions 3,240 to 3,395 cm^−1^ indicate the presence of hydroxyl groups (-OH) free stretch as a result of hydrogen bonding and N-H str modes. The observed peaks in the regions (2,930 to 2,860 cm^−1^ and 2,361 cm^−1^) could be ascribed to aliphatic, strongly symmetrical, and asymmetrical vibrations of CH_3_, CH_2_, and -C-H- in lipids. The bands at 1,646 cm^−1^ appeared due to the –CH_2_ group, which indicates the presence of various functional groups of lipid and polysaccharide components. The peak was observed at 1,117 cm^−1^, affirming the presence of C-O-C group vibrations in the cyclic structures of polysaccharides. Furthermore, the bands detected in the 904 cm^−1^ region correlated with the glycosidic linkage type. Similar FTIR data were found by Deepika et al. (2017) and Sabarinathan et al. (2021). The FTIR profile authenticated that the produced biosurfactant consists of hydrophobic chains that comprise lipids, sugars, and hydrophilic glycolipids.

### 3.9 Biological activities of the produced biosurfactant

#### 3.9.1 Antimicrobial activity

**Figure 6:**
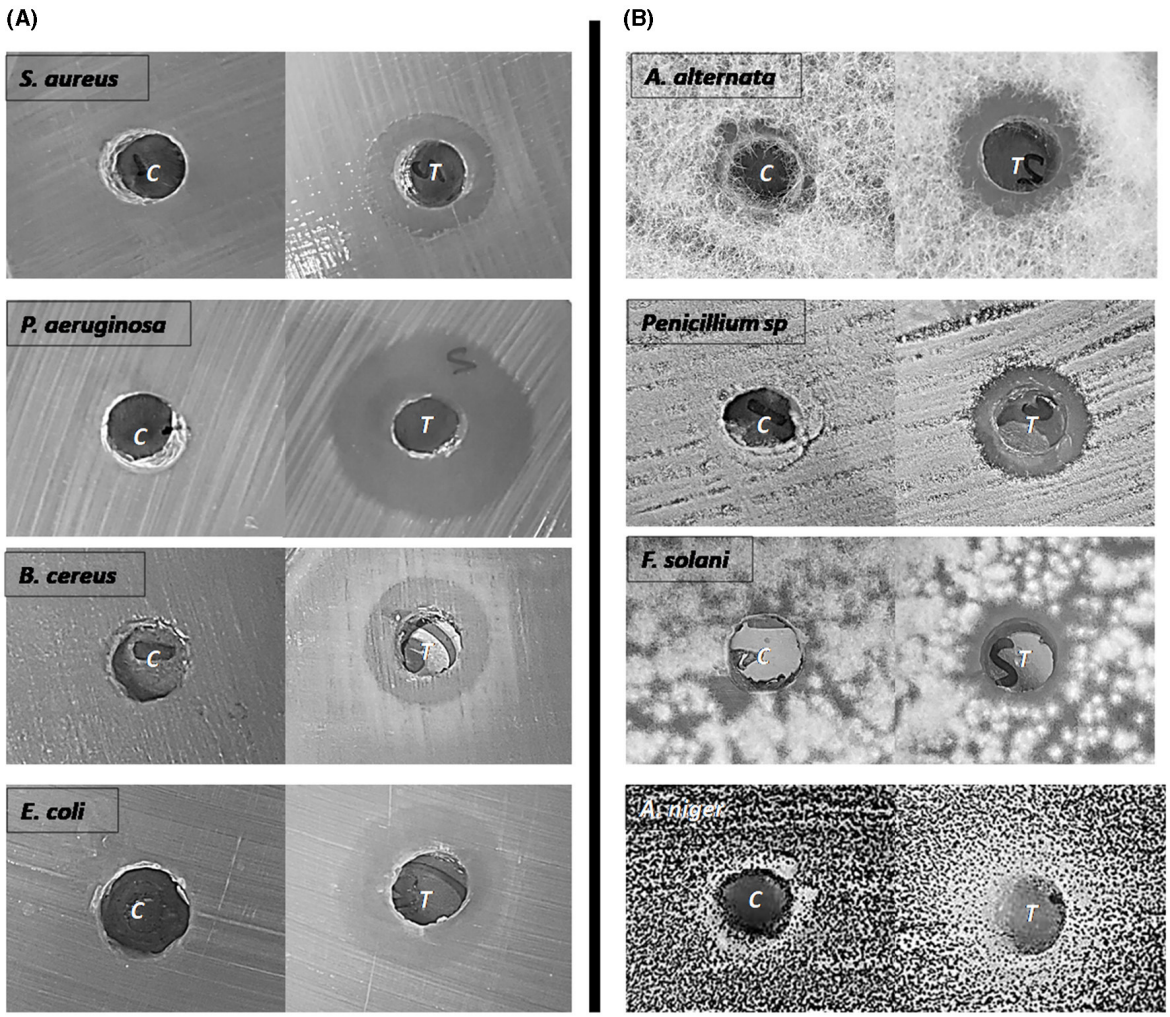
Antibacterial **(A)** and Antifungal **(B)** activity of biosurfactants. The inhibition zone diameter was measured against MBM as a control.

The antimicrobial activity of the biosurfactants produced by HA-2 against the tested pathogenic bacteria and fungi was assessed using the well diffusion method. HA-2 had antibacterial effects with maximum inhibition zone 20.6 ± 3.7 mm with *P. aeruginosa*, followed by *E. coli* (18.3 ± 1.1 mm), *B. cereus* (15.3 ± 0.5 mm), and *S. aureus* (14.67 ± 1.5 mm; Table 2 and Figure 6).

**Table 2:** Antimicrobial activity of biosurfactants produced by thermophilic HA-2.

**Treatment**	**Zone of inhibition (mm)**			
	***P. aeruginosa***	***B. cereus***	***S. aureus***	***E. coli***
HA-2 biosurfactant	20.6^a^ ± 3.7	15.3^a^ ± 0.5	14.67^a^ ± 1.5	18.3^a^ ± 1.1
	***A. niger***	***F. solani***	***Penicillium*** **sp**.	***A. alternata***
	18.6^a^ ± 1.5	15^a^ ± 0	13.67^a^ ± 0.5	16.3^a^ ± 1.1
Control MBM	0	0	0	0

Data are represented as mean standard deviations of values from triplicate experiments.

^a^Indicates significant variation when compared to control.

Regarding the antifungal activity, HA-2 had antifungal effects with inhibition halos 18.6 ± 1.5 mm with *A. niger*, 16.3 ± 1.1 mm with *A. alternata*, 15 ± 0.0 mm with *F. solani*, and 13.67 ± 0.5 mm with *Penicillium* sp. (Table 2 and Figure 6).

#### 3.9.2 Antioxidant activity

The biosurfactant produced by thermophilic AH-2 displayed high antioxidant activity of 90.4%.

#### 3.9.3 Effect of the produced biosurfactant on *T. aestivum* growth

To investigate the impact of biosurfactants produced from the isolated AH-2 on wheat (*T. aestivum)* grain germination and growth, the grains were watered with 2, 5, and 10% concentrations of the biosurfactant. It was noticed that the concentrated biosurfactant completely inhibited grain germination. Therefore, the previous concentrations were evaluated as they induced complete germination. The results in Table 3 showed that all the investigated concentrations of biosurfactants significantly (*P* < 0.05) increased radical length when compared with MBM or distilled water. The number of seminal roots was not significantly affected by any treatment. On the other hand, the grains that were watered with 2% of the biosurfactant exhibited significant (*P* < 0.05) improvement in coleoptile length, longest seminal roots, and seedling dry biomass with 32.6 ± 1.8, 31.7 ± 3.7, and 36.7 ± 1.1, respectively, when compared with the negative control (distilled water). These results affirmed that 2% of the biosurfactant was the optimum concentration for enhancement of wheat grain germination and seedling health.

**Table 3:** Impact of biosurfactants on growth of *Triticum aestivum*.

**Treatment**	**Biosurfactant (%)**	**Radical length (mm)**	**Coleoptile length (mm)**	**Longest seminal roots (mm)**	**Number of seminal roots**	**Dry biomass (mg)**
AH-2	**2**	25.3^a, b^ ± 1.8	32.6^a, b^ ± 3.7	31.7^a, b^ ± 4.8	4 ± 0.3	36.7^a, b^ ± 1.1
	**5**	23.5^a, b^ ± 1.3	24.3 ± 5.3	24.4 ± 5.53	4 ± 0.5	26.3^a^ ± 3.01
	**10**	23.7^a, b^ ± 4.6	30.2 ± 7.3	25.7 ± 6.2	4 ± 0.4	23.2^a^ ± 1.3
Dist. water	**-**	14.9^a^ ± 4.2	29.5 ± 8.01	22.7 ± 7.2	4 ± 0.1	33.1 ± 4.08
Control MBM	**-**	19.8^b^ ± 2.8	28.1 ± 8.9	25.3 ± 7.2	4 ± 0.0	25.7 ± 2.65

Data represented as mean ± standard deviations of values from duplicate experiments.

^a, b^Indicate significant difference against dist. water and MBM, respectively.

Bold values indicate the concentration of biosurfactant (%).

## 4 Discussion

Microorganisms isolated from extreme environments have significant potential for biotechnological applications, particularly biosurfactants, which are a promising sustainable alternative to chemical surfactants. Therefore, substantial efforts have been made to investigate bioproduct synthesis under extreme conditions (Schultz and Rosado, 2020). Uhud mountain is an arid region in Saudi Arabia that is characterized by drought and high temperature of 60°C during summer. This mountain is formed from volcanic rock including red, black, and dark green granite. Interestingly, this geographical area has not been microbiologically explored before. In the current study, sampling and isolation of thermophilic biosurfactant-producing bacteria were performed based upon prior enrichment of the collected bacteria samples. The enrichment method is an effective method for isolation of microorganisms adapted to harsh conditions, as mentioned by Govil et al. (2020). Many studies have reported that the biosurfactant-producing bacteria are ubiquitous in nature and mostly found in hydrocarbon-polluted areas as well as in volcanic zones, hot springs, and other sites (Schultz and Rosado, 2020). Joy et al. (2017) isolated 11 biosurfactant-producing thermophilic bacteria, such as *Bacillus, Citrobacter, Lysinibacillus, Ochrobactrum*, and *Pseudomonas*, from hot spring water and desert soil.

The thermophilic bacteria were grown on MBM supplemented with WSFF oil. Frying is the most popular process of food preparation in the food industry and also in the household, which generates large amount of disposal frying oil, hence contributing to the pollution of the environment (Wadekar et al., 2012). Many literature studies have suggested that the reuse of waste frying oil has significant potential to develop alternative and economic substrates for rhamnolipids and sophorolipids biosurfactant production (Pathania and Jana, 2020; Pathania et al., 2021). Moreover, *Candida bombicola* and *Starmerella bombicola* produced sophorolipid surfactants (Wadekar et al., 2012), three different *Bacillus subtilis* strains produced surfactin (Valenzuela-Ávila et al., 2020), *Bacillus licheniformis* NJ1 produced lipopeptide biosurfactant (Pathania et al., 2021), and *Pseudomonas aeruginosa* produced rhamnolipids (Deepika et al., 2017; Pathania and Jana, 2020) via hydrolysis of waste frying oil. *G*. *stearothermophilus* UCP 986 generated biosurfactants in a BioFlo fermentor at 45°C, 32 h, and 300 rpm, using optimum growth medium consisting of palm oil (7.5%) and maize steep liquor (4.5%; Jara et al., 2013). In another investigation, *G. stearothermophilus* DSM2313 with pH = 7, glucose = 50 g/L, and NH_4_NO_3_ = 2 g/L was also shown to produce biosurfactants (Czinkóczky and Németh, 2023). Bio-emulsifiers generated from *G. stearothermophilus* strain had 71.4% sugar and 27.8% protein. Many hydrocarbons were emulsified by the bioemulsifier at varying salinity, temperature, and pH (Zhou et al., 2016). Zhou et al. (2018) isolated *Geobacillus stearothermophilus* A-2 from a high-temperature petroleum reservoir. The strain had the ability to degrade hydrocarbons and produced a bio-emulsifier.

The thermophilic isolates were screened for biosurfactant production by the hemolytic test, CTAB test, oil displacement test, drop collapsing method, and emulsification index test. The hemolytic activity of the supernatants was similar to that observed in the study performed by Ibrahim (2018). They documented the presence of a correlation between biosurfactant production and the hemolytic zones. Zaragoza et al. (2010) reported that biosurfactants cause hemolysis through enhanced permeability of the biological membrane as well as altering their structure and function. However, Satpute et al. (2008) recommend the use of combination methods rather than the single method. The oil collapse and oil spreading tests showing similar results to Sumathi and Yogananth (2016) and Nayarisseri et al. (2018). Furthermore, Li H. et al. (2023) suggested that the oil spreading area in the oil displacement assay is more related to the concentration of the biosurfactants present in the culture. The increased accumulation of the surfactant contributes to the decrease in surface tension, thus reducing the repulsive forces between the two different phases and allowing easy mixture. The biosurfactant of the bacterial isolates showed high emulsification activity, and these results were in agreement with the results obtained by Nwaguma et al. (2016) and Sumathi and Yogananth (2016). They found that biosurfactants produced by the *Klebsiella pneumonia* strain IVN51 and the *Pseudomonas aeruginosa* strain revealed maximum emulsification indices of 60 and 52%, respectively. This finding may be attributed to their amphiphilic configuration; biosurfactants allow the dispersion of hydrophobic substances in the aqueous solution by increasing the solubility of hydrophobic substances as well as reducing the interfacial tension between immiscible liquids (Jahan et al., 2020). The anionic surfactant was detected by the CTAB assay, whereas the cationic CTAB interacts with the anionic surfactant, which is confirmed by the formation of blue haloes, as reported in many studies (Sidkey et al., 2016; Ibrahim, 2018).

Additionally, the production of rhamnolipid, an anionic glycolipid biosurfactant, by the isolate was confirmed via PCR of *rhlAB*. Kumar et al. (2008) reported that both the CTAB assay and *rhl* detection denote the synthesis of rhamnolipids by *P. aeruginosa* DHT2. Biosurfactants are categorized into high- and low-molecular weight groups. High-molecular weight biosurfactants comprise particulate and polymeric biosurfactants, whereas low-molecular weight biosurfactants include glycolipids, phospholipids, and lipopeptides (Venkataraman et al., 2022). The preliminary characterization of biosurfactants revealed that the produced biosurfactant in this study contains carbohydrate groups, which was consistent with the results reported by Zhang et al. (2012). They reported that biosurfactants produced by *P. aeruginosa* include a group of carbohydrates classified as glycolipids using the same test.

The optimization of biosurfactant production by the isolate HA-2 was determined by changing one factor each time. The highest emulsification activity was obtained at pH = 9, and it decreased when the pH declined. The results of pH were consistent with that obtained by Gumaa et al. (2010). They reported maximum biosurfactant production at pH = 9 with *Serratia marcescens* N3 and *Klebsiella pneumonia* IVN51. Elazzazy et al. (2015) also reported that the production of biosurfactants by *Virgibacillus salarius*was optimum at pH = 9. It was also reported that biosurfactant production by *B. subtilis* was optimum in the pH range of 6–10 (El-Sersy, 2012). Furthermore, biosurfactants produced at a high temperature range are more favorable for biotechnological applications (Karadayi et al., 2021). The current study revealed that the optimum biosurfactant production of thermophilic AH-2 was recorded at 50°C, encouraging the application of this strain in many biotechnological applications.

Regarding the impact of different carbon and nitrogen sources, the biosurfactants produced by the bacterial isolates were successfully used in emulsifying oil with different emulsification degrees. The highest emulsification value was recorded when potato peel was used as a carbon source. Potato peels are a rich nutrient source as they contain high carbohydrate and protein contents, which enhance bacterial growth. Moreover, the highest induction of biosurfactants on potato peels may be attributed to the phenolic compounds that are present in it (Sampaio et al., 2020). These results were supported by those of Ansari et al. (2014) and Kumar et al. (2016), who reported high emulsification activity of the produced biosurfactant of 70 and 65.5% via hydrolyzing of potato peels as a carbon substrate. The significant decrease in the biosurfactant production with orange peel supplementation may be attributed to the presence of essential oils that exhibit antibacterial activity (Geraci et al., 2017). It may retard the bacterial growth and subsequently suppress the induction of biosurfactants via AH-2 (Geraci et al., 2017).

Moreover, nitrogen plays a significant role in biosurfactant production. The results indicated that no significant variation was observed between the investigated nitrogen sources; however, the optimum emulsification activity was determined with yeast extract supplementation. This finding is consistent with the findings of Saimmai et al. (2013) and Akbari et al. (2021). These authors reported high biosurfactant production by *Kocuria rosea* ABR6 and *Inqulinus limosus* KB3 with yeast extract supplementation. Additionally, yeast extract in low concentrations increasingly favored biosurfactant production, as it was utilized for cell growth rather than biosurfactant production (Fontes et al., 2010).

As the concentration of surfactant increases, surface tension steadily decreases. This decline ends at a concentration known as the critical micelle concentration (CMC). It is a crucial parameter for determining the interfacial activity of surface active agents (Zhou et al., 2019). The surface tension is nearly constant above the CMC. Biosurfactants have CMC values ranging widely from 5 to 386 mg/L (Abbasi et al., 2012). By using waste from soybean oil refineries, Abalos et al. (2001) obtained CMC values of 106, 150, and 234 mg/l for various combinations of mono-rhamnolipids and di-rhamnolipids. Recently, the isolated surfactin from *Geobacillus thermodenitrificans* ME63 was 55 mg/L (CMC), and the surface tension at CMC was 35.9 mN/m (Li J. Y. et al., 2023).

The antimicrobial activity of the biosurfactants against *P. aeruginosa, E. coli, B. cereus, S. aureus, A. niger, A. alternata, F. solani*, and *Penicillium* sp. was studied. The biosurfactant produced by the bacterial isolates exhibited antibacterial and antifungal activity, with similar results to that reported by Matei et al. (2017) and Aguirre-Noyola et al. (2020). They found that the extracted biosurfactants exhibited antibacterial and antifungal activities. This may be attributed to the interaction of biosurfactants with membrane phospholipids, lead to changes the membrane permeability and alters the biological functions (Adetunji et al., 2022). The DPPH scavenging activity of the rhamnolipid biosurfactant extracted in this study suggested the biotechnological role it plays in different fields. The DPPH scavenging activity exhibited an increase with the concentration of the biosurfactants produced by various *Bacillus* spp., as previously reported by Yalcin and Cavusoglu (2010) and Ciurko et al. (2022). Therefore, the extracted biosurfactant in this study is suggested to be a valuable product owing to its antibacterial, antifungal, and antioxidant activities.

In addition, rhamnolipid biosurfactants extracted from AH-2 exhibited *in vitro* enhancement of wheat plant growth. The low concentration (2%) of biosurfactants exhibited significant improvement in plant growth. It was reported that biosurfactants enhance the germination process by improving the permeability, which increases nutrient diffusion (Umar et al., 2021). Other studies suggest that biosurfactants may promote plant growth by increasing the resistance of plants to pathogenic microbes (Sachdev and Cameotra, 2013). The weak impact of biosurfactant on plant growth recorded at a high concentration of surfactants may be because the high concentration of biosurfactants may cause damage to plant tissues or impede plant development by augmenting the concentration of hydrophobic compounds in the environment, which could not be assimilated by rhizosphere microorganisms or through the release of some compounds from soil, which results in growth inhibition (Marchut-Mikołajczyk et al., 2021).

It could be concluded that a thermophilic strain HA-2 was isolated from Uhud mountain and showed high capability of producing a rhamnolipid biosurfactant by use of waste sunflower frying oil. Carbon sources, nitrogen sources, pH, and incubation temperature all influence the production of biosurfactants. The produced biosurfactant exhibited promising antioxidant and antimicrobial activities against different bacterial and fungal strains. In addition, it showed positive results on plant growth, showing the possibility of using them in agricultural applications. The ability to produce biosurfactants from thermophilic bacteria using low-cost materials makes them interesting options not only economically but also environmentally. Additionally, this study revealed that the Uhud mountain has a rich diversity of microorganisms with potential and a broad range of industrial, agricultural, and medical applications.

## Data availability statement

The datasets presented in this study can be found in online repositories. The names of the repository/repositories and accession number(s) can be found at: https://www.ncbi.nlm.nih.gov/, OR911984.

## Author contributions

HA: Supervision, Writing—original draft, Writing—review & editing. AAA: Data curation, Methodology, Writing—original draft. AA: Data curation, Formal analysis, Writing—original draft. DB: Visualization, Writing—review & editing. MW: Data curation, Visualization, Writing—review & editing. AM: Conceptualization, Writing—original draft, Writing—review & editing.
